# Study of MicroRNA-192 as an Early Biomarker for Diagnosis of Diabetic Nephropathy

**DOI:** 10.3390/diagnostics15121504

**Published:** 2025-06-13

**Authors:** Mohamad Motawea, Mayada S. Khalel, Ismail Kandil, Ahmed Mohsen Faheem, Maysaa El Sayed Zaki, Mostafa Abdelsalam, Fady Kyrillos

**Affiliations:** 1 Diabetes and Endocrinology, Faculty of Medicine, Mansoura University, Mansoura 35516, Egypt; fadyazmy@mans.edu.eg; 2 Internal Medicine Department, Faculty of Medicine, Mansoura University, Mansoura 35516, Egypt; dr_mayada@mans.edu.eg; 3 Geriatric Medicine Unit, Internal Medicine Department, Faculty of Medicine, Mansoura University, Mansoura 35516, Egypt; i.kandil843@gmail.com; 4 Department of Medical Biochemistry and Molecular Biology, Faculty of Medicine, Mansoura University, Mansoura 35516, Egypt; dr_ahmed1986@yahoo.com; 5 Clinical Pathology, Faculty of Medicine, Mansoura University, Mansoura 35516, Egypt; may_s@mans.edu.eg; 6 Mansoura Nephrology and Dialysis Unit, Internal Medicine Department, Faculty of Medicine, Mansoura University, Mansoura 35516, Egypt; darsh1980@mans.edu.eg; 7 Alameen General Hospital, Taif 26511, Saudi Arabia

**Keywords:** diabetic nephropathy, DM type 2, MicroRNA-192, albuminuria, eGFR

## Abstract

**Background/Objectives:** Diabetic nephropathy (DN) is a serious complication of diabetes mellitus. This clinical condition is diagnosed through the detection of microalbuminuria. Molecular biomarkers such as MicroRNA-192 may play a role in the early diagnosis of this condition. This study aims to compare the serum concentrations of MicroRNA-192 in diabetic patients with and without DN and in healthy individuals. **Methods:** This study was a retrospective case-control study that included three groups. Group I included diabetic patients without DN, Group II included patients with DN, and Group III included healthy control subjects. Blood samples were obtained from each participant and subjected to a full biochemical study including creatinine, albumin, and the detection of MicroRNA-192 by real-time polymerase chain reaction. **Results:** There were significant differences among the MicroRNA-192 levels in the three groups (*p*-0.001). There was a significant increase in the MicroRNA-192 level in Group I (1.35 ± 7 0.5) compared with Group II (0.65 ± 7 0.2, *p*3 = 0.001) and Group III (0.83 ± 7 0.3, *p*1-0.001). There was a significant reduction in the MicroRNA-192 level in Group II compared with Group III (*p*2-0.001). **Conclusions:** This study highlights the potential role of serum miR-192 as a noninvasive biomarker for the early detection of DN in patients with type 2 DM. Our findings demonstrated that serum miR-192 levels were significantly reduced in patients with DN compared with diabetic patients without nephropathy and healthy controls, suggesting the possible protective role of miR-192 in early disease stages.

## 1. Introduction

Diabetic nephropathy (DN) is a prevalent consequence of diabetes mellitus, occurring in approximately 9% of people with either type 1 or type 2 diabetes mellitus (type 2 DM) [1]. DN is one of the leading causes of end-stage renal disease [2,3]. The DN histological hallmark is an extracellular deposition of proteins such as laminin, fibronectin, and collagen that leads to the thickening of glomerular basement membranes (GBM) and mesangial expansion [4].

The first phase of DN is GBM thickening, and it is associated with a normal glomerular filtration rate (GFR) without albuminuria. In the second phase, there is mild to severe mesangial expansion. It usually occurs 2 years from the first stage with still-normal GFR and no significant clinical manifestations. The third phase is glomerulosclerosis and the formation of Kimmelstiel–Wilson nodules (nodular sclerosis) with urinary microalbumin (30–300 mg /day). This stage starts 5 to 10 years after the GBM onset. The fourth phase is advanced diabetic glomerulosclerosis with urinary microalbumin of more than 300 mg/day. The ESDR is total kidney failure with a GFR below 15 mL/min/1.73 m^2^ [5,6,7].

In DN, many pathways are activated, such as renin–angiotensin–aldosterone, inflammatory and profibrotic cytokines as tumor necrosis factor-α (TNF-α) and transforming growth factor β (TGF-β), as well as several miRNAs such as miR-192, miR-21, miR-29, miR-342, and miR-214 [8,9,10].

There is a need for expeditious and noninvasive techniques to identify this advancing DN. Scientists have acknowledged the involvement of microRNAs (miRNAs) in the pathological mechanism of DN. MiRNAs are small, endogenous, noncoding RNA molecules that control gene expression through processes happening after transcription, specifically by making it easier for mRNA to break down or stop translation [11,12]. Therefore, miRNAs control a wide range of biological processes in cells, such as cell growth, cell specialization, programmed cell death, apoptosis, and passive cell death. 

MiRNA-192 (miR-192) is encoded by a gene located on chromosome 11. Two fully developed transcripts, miR-192 (miR-192-5p) and miR-192* (miR-192-3p), constitute it [13]. Differentiation, apoptosis, proliferation, epithelial–mesenchyme transition, angiogenesis, metabolism, oxidative stress, inflammatory responses, and drug resistance are some of the physiological and pathological processes that MiR-192 controls [14,15]. Inhibiting the miR-192-targeted genes leads to various effects, such as the degradation of mRNA and the suppression of protein translation. A range of ailments, including respiratory, digestive, circulatory, and urinary system disorders, are linked to the dysregulation of miRNA-192.

The renal cortex is the primary location of miRNA-192 (miR-192), suggesting its potential role in the development of DN [8].

There have been mixed results from different studies about the levels of miR-192 in blood, urine, and kidney tissue from people with different stages of DN. These data suggest that the serum’s concentration of miR-192 can serve as an early diagnostic marker for DN. A higher level of miR-192 in the later phases may indicate a deterioration of DN [8]. So, the point of this study was to compare the amount of miR-192 in the blood of diabetic people with and without DN in addition to comparison with a healthy control group.

## 2. Materials and Methods

This retrospective case-control study enrolled patients from the outpatient clinics of the Mansoura University Hospital in Egypt between January 2022 and March 2024. This study also used age- and sex-matching controls. The time-nonprobability sampling method, which relied on the study period during which the recruited patients met the inclusion criteria for DN staging, determined the patient sample size.

The patients’ eligibility criteria were that they were adults aged 18 or older, had no previous history of renal problems, and had type 2 DM. This study excluded patients with the following criteria: cardiovascular disease, renal illnesses caused by other factors such as autoimmune disorders or malignancies, hypertension, heart diseases, cancer, and pregnancy. We categorized the patients according to their albuminuria levels and estimated glomerular filtration rates (eGFR). Group I consisted of diabetic patients with normal albuminuria levels below 30 mg/g and eGFRs higher than 60 mL/min/1.73 m^2^. Group II, on the other hand, included diabetic patients with DN characterized by albuminuria levels over 30 mg/g and eGFRs lower than 60 mL/min/1.73 m^2^ [16]. Group III consisted of age- and sex-matched healthy adult individuals as controls, who had normal fasting blood glucose levels below 100 mg/dL and who did not currently use any chronic medications, had no history of kidney diseases, and had normal results for kidney function testing. Every participant underwent comprehensive medical history and physical assessments. 

We conducted diagnostic examinations to analyze and evaluate many aspects of a subject’s medical or chemical composition.

### 2.1. Laboratory Study

We obtained a blood sample and a urine sample from each participant using meticulous aseptic techniques, and we also collected an early-morning midstream urine sample from each and stored it in a sterile container. After isolating the serum, we used it to determine the concentration of creatinine. To separate the urine, we used centrifugation and collected the liquid that was above the sediment. Then, we used the Cobas s201 system (made by Roche Diagnostics, 9115 Hague Road, P.O. Box 50457, Indianapolis, IN, USA) to check the levels of albumin and creatinine in the urine. We stored the serum at a temperature of −80 °C until we conducted our investigation on micRNA-192. The Cobas C system employs the immunoturbidimetric technique (mg/L) for quantifying urine albumin. The g/L Cobas 201s system, manufactured by Beckman Coulter, utilizes the Jaffe method for quantifying urine creatinine levels. We employed the Cockcroft–Gault formula, developed in 1973, to calculate the eGFR. The eGFR is determined using the following formula: eGFR = {(140 − age) × weight/(72 × SCr)} × 0.85 (for females). The term SCr refers to serum creatinine.

#### 2.1.1. Purification of RNA and Reverse Transcriptase

The RNA was isolated from the serum samples using the Direct-zol RNA Miniprep Plus kit (Cat # R2072, Zymo Research, Irvine, CA, USA). 

We conducted reverse transcription on the extracted RNA from the samples using reverse transcriptase and real-time PCR.

#### 2.1.2. Real-Time PCR

The experiment employed real-time quantitative polymerase chain reaction (qPCR), with the primer sequences for miR-192 being 5′-GCGGCGGCTGACCTATGAATTG-3′ and 5′-ATCCAGTGCAGGGTCCGAGG-3′. The primer sequences for U6 were 5′-TCCGATCGTGAAGCGTTC-3′ and 5′-GTGCAGGGTCCGAGGT-3′. The reaction was conducted by subjecting it to incubation at 50 °C for 2 min, followed by subsequent incubation at 95 °C for 10 min. Subsequently, we performed a cyclic reaction, alternating between a temperature of 95 °C for 10 s and a temperature of 60 °C for 60 s. We iterated this cycling procedure for a cumulative total of 45 rounds. We utilized ABI Company’s ABI PRISM 7900 system for analysis. The expression level of miR-192 was quantified using the 2 Ct methods, with U6 acting as the internal reference [17,18].

#### 2.1.3. Statistical Analysis

The data were analyzed by SPPS24. The normality test used was the Q-Q plot. All three Q-Q plots showed that the data were normally distributed in all three groups. This was confirmed by skewness and kurtosis z-scores between −2.58 and +2.58 for all groups. The numerical data were expressed as mean and standard deviation (SD), and comparison was performed by ANOVA test. The homogeneity of variances was violated as assessed by the Levene test (*p* < 0.001). Welsh ANOVA was reported, and Games–Howell Post Hoc tests were used for pairwise comparisons. *p* was considered significant if below 0.05. Receiver operator characteristics (ROC) were used to evaluate the diagnostic accuracy of different markers in patients with DN using MedCalc^®^ Statistical Software, version 23.1.5 [19]. The P value was considered statistically significant when less than 0.05.

##### Sample Size

As most clinical trials indicated that miR-192 might play a protective role in DN development and progression, such as the one performed by Wan et al. [8], we hypothesized a large effect size (Cohen’s f = 0.4) when comparing miR-192 among the control subjects, the diabetics without DKD, and the diabetics with DKD. In a one-way ANOVA study, a sample size of 50 subjects was obtained from each of the three groups. The total sample size of 150 participants achieved a 99% power to detect a large effect size (Cohen’s f = 0.4) in miR-192 among the three groups at a statistical significance of 0.05 (α = 0.05).

## 3. Results

This study included 150 subjects classified into three groups: Group I included diabetic patients without DN, Group II included diabetic patients with DN, and Group III included age- and sex-matched healthy control subjects. There was a significant increase in fasting blood glucose, HB1c, blood urea nitrogen (BUN), creatinine, and albumin in the urine in Groups I and II compared with Group III (*p*-0.001). The duration of diabetes was significantly longer in group II (7.7 ± 3.3 years) compared with Group I (5.23 ± 2.8, *p*-0.001). The eGFR was significantly lower in Groups I and II compared with Group III (*p*-0.001). Also, the albumin/creatinine ratio had significantly increased values in Groups I and II compared with Group III (*p*-0.001) (Table 1).

**Figure 1:**
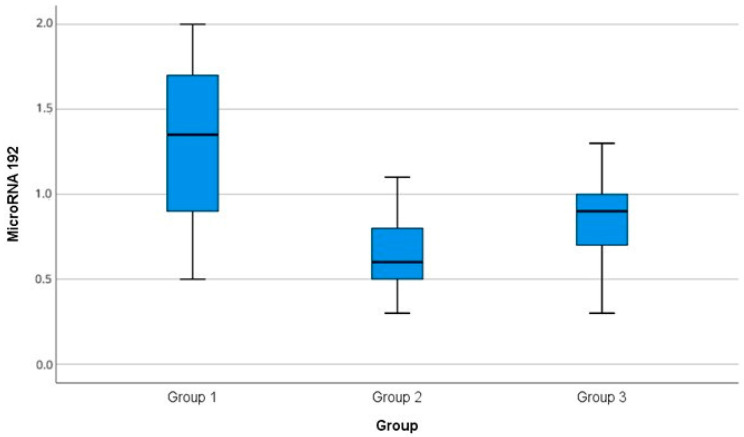
Boxplot for MicroRNA 192 in the three groups.

There was a significant difference among the miR-192 serum levels in the three groups (*p*-0.001). There was a significant increase in miR-192 level in Group I (1.35 ± 0.5) compared with Group II (0.65 ± 0.2, *p*3-0.001) and Group III (0.83 ± 0.3, *p*1-0.001). There was a significant reduction in the miR-192 level in Group II compared with Group III (*p*2-0.001), as shown in Table 2, Figure 1. The P among the groups was determined via ANOVA test.

The post hoc test included the following:

*p*1 between control and diabetic patients without DN;
*p*2 between the diabetic patients with DN and the control;
*p*3 between the diabetic patients without and with DN.

Table 3 summarizes the correlations among MicroRNA-192 levels and various clinical and biochemical parameters in the studied subjects. There was a significant negative correlation between creatinine, BUN, urinary albumin, urinary albumin/creatinine ratio, and miR-192 level (*p*-0.001). There was a significant negative correlation between the duration of diabetes and miR-192 (*p*-0.001). There was a significant positive correlation between eGFR and miR-192 (*p*-0.001) (Table 3).

**Figure 2:**
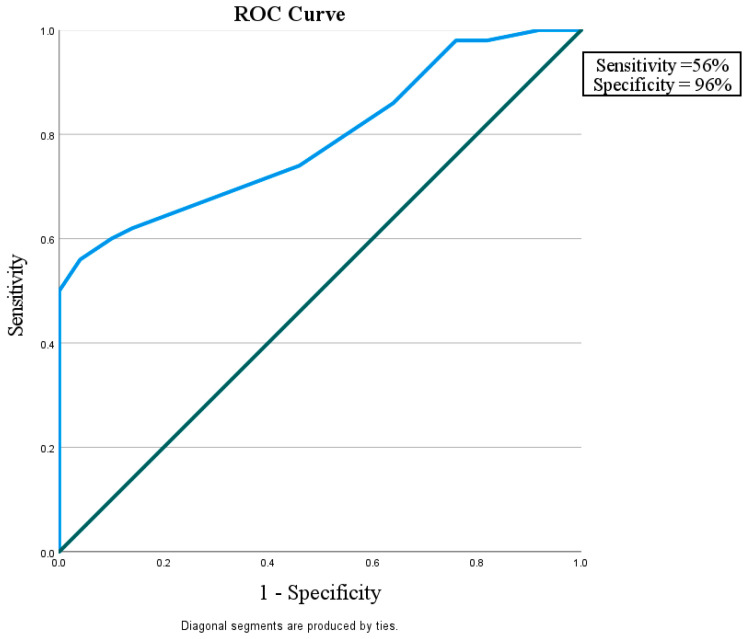
ROC curve for the MicroRNA 192 cutoff value to discriminate between Group I (DM without DN) and Group III (Control).

**Figure 3:**
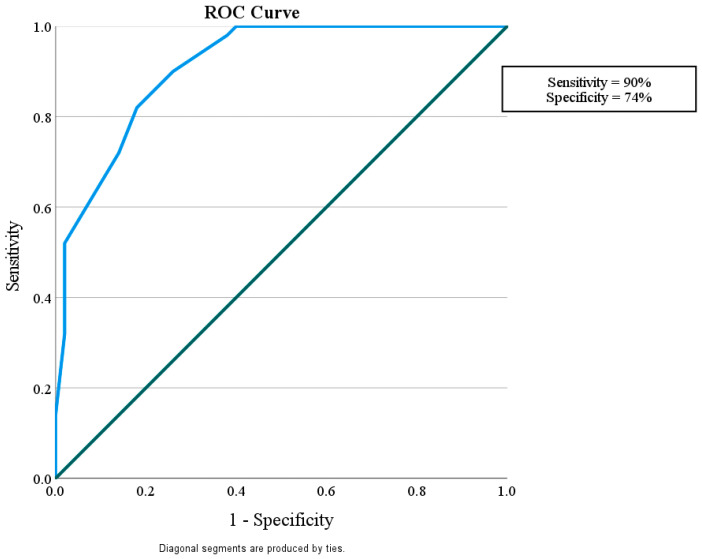
ROC curve for the MicroRNA 192 cutoff value to discriminate between Group II (DM with DN) and Group I (DM without DN).

**Figure 4:**
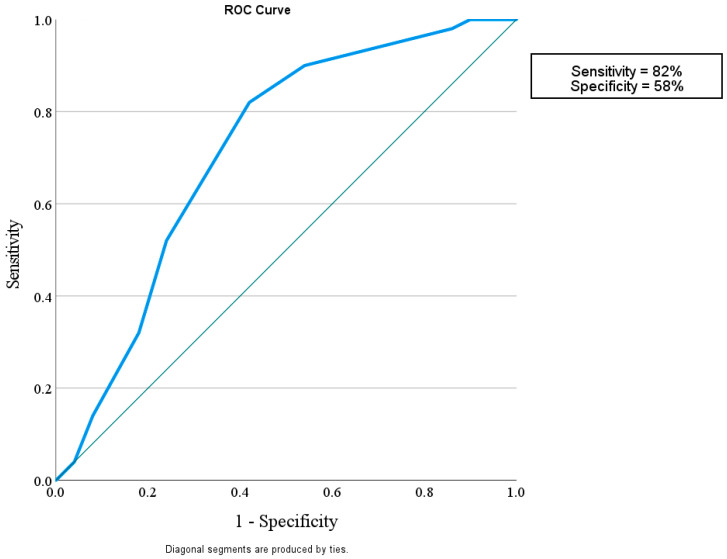
ROC curve for the MicroRNA 192 cutoff value to discriminate between Group II (DM with DN) and Group III (Control).

This study considered how well miR-192 could play a role in protection against nephropathy in diabetic patients without albuminuria and with eGFR > 60 mL/min/1.73 m^2^ in comparison with control subjects with a sensitivity of 56% and a specificity of 96%. It also showed that miR-192 could tell the difference between diabetic patients with or without nephropathy, with a sensitivity of 90% and a specificity of 74%. Finally, miR-192 could distinguish the difference between diabetic patients with DN and the control group with a sensitivity of 82% and a specificity of 58% (Figure 2, Figure 3 and Figure 4).

Area under the ROC curve (AUC)
Area under the ROC curve (AUC) 0.790Standard Error ^a^0.045295% Confidence interval ^b^0.697 to 0.865z statistic6.419Significance level P (Area = 0.5)<0.0001^a^ DeLong et al. [20]. ^b^ Binomial exact.


Youden index
Youden index J0.5200Associated criterion>1.2

Area under the ROC curve (AUC)
Area under the ROC curve (AUC) 0.911Standard Error ^a^0.027395% Confidence interval ^b^0.837 to 0.959z statistic15.050Significance level P (Area = 0.5)<0.0001^a^ DeLong et al. [20]. ^b^ Binomial exact.


Youden index
Youden index J0.6400Associated criterion≤0.9

Area under the ROC curve (AUC)
Area under the ROC curve (AUC) 0.717Standard Error ^a^0.052195% Confidence interval ^b^0.618 to 0.802z statistic4.158Significance level P (Area = 0.5)<0.0001^a^ DeLong et al. [20]. ^b^ Binomial exact.


Youden index
Youden index J0.4000Associated criterion≤0.8

## 4. Discussion

This study was conducted to assess the serum level of miR-192 in patients with type 2 DM to investigate its significance as a biomarker for the early diagnosis of DN.

MiR-192 is highly expressed in the kidney renal cortex. Many studies have confirmed that miR-192 has an important role in kidney fibrosis, but the conclusions about the effect of miR-192 in DN are still controversial. Studies about miR-192 expression in serum and urinary excretion show conflicting results [8,11].

In the present study, Group II (diabetic patients with DN) had significantly lower levels of miRNA-192 (0.65 ± 0.2) in comparison with the diabetic normoalbuminuric Group I (1.35 ± 0.5), (*p*3-0.001). Additionally, Group II had a significantly lower level of miRNA-192 (0.65 ± 0.2) in comparison with the nondiabetic control, Group III (0.83 ± 0.3) (*p*2-0.001).

A similar result was reported by Ma et al. [11], who conducted a large cohort study that included 464 diabetic patients and a control group of 127 normal, healthy patients; they reported that miR-192 expression was decreased significantly in both the patients with microalbuminuria and macroalbuminuria in comparison with those with normoalbuminuria (*p* < 0.01). They suggested that miR-192 may be a potential marker for diagnosis of DN.

Akpınar et al. [21] found that the serum expression of miR-192-5p in the DN group was significantly lower than in the control group (*p*-0.027), and the area under the curve value was 0.717 compared with the control group, suggesting miR-192’s role in the development of DN.

Yang et al. [22] conducted a study on 283 DN patients to investigate miR-192 and found that there was a statistically significant decrease in the miR-192 serum levels and an increase in urine excretion in patients with DN. The authors suggested that both urinary and serum miR-192 could be potential biomarkers of DN.

Al-Kafaji et al. [23] conducted a study that included a total number of 85 patients divided into three groups: a microalbuminuric group (*n* = 15), a macroalbuminuric group (*n* = 10), and a normoalbuminuric group (*n* = 30). They found that there was a statistically significant 19-fold decrease in the expression of miR192 in the macroalbuminuric group compared with the normoalbuminuric group (*p* < 0.005), and the expression decreased by 2.4-fold in the microalbuminuric group in comparison with the normoalbuminuric group, but it was statistically insignificant (*p* > 0.05). They suggested that the miR-192 serum level may be used as a biomarker for the early prediction of DN in patients with type 2 DM.

Abou Eleila et al. [24] conducted a cross-sectional study that included 50 patients with type 2 DM and was divided into three groups: Group I included patients with normoalbuminuria (*n* = 16), Group II included patients with microalbuminuria (*n* = 16), and Group III included patients with overt proteinuria (*n* = 18). They assessed the correlation between the serum miR-192 and DN. Regarding the serum miR-192 level, the serum level decreased with diabetes progression, but no statistically significant difference was detected among the three groups (*p*-0.225). This result could be attributed to the small sample size, potential differences in methodology, population characteristics, or other influencing factors.

Different results were reported by Chien et al. [25], who found that, regarding miR-192 expression, there was no significant difference between type 2 DM patients with or without DN. However, the level was significantly higher in the overt proteinuria groups compared with the microalbuminuria group (*p*-0.0138), which is contrary to all other studies. In this study, all patients with DN received ARBS or ACEIs unless contraindicated. These medications may affect miRNA-192 levels.

Ebadi et al. [26] conducted an experimental study to investigate the effects of applying captopril, spironolactone, and their combination on renal performance indices and microRNAs expression. They found that applying both captopril and spironolactone improve DN by targeting and changing the expressions of miR-192 and the miR-29 family.

Another experimental study conducted by Yu et al. [27] investigated the effects of metformin on DN mice. They found that the expressions of miR-192 increased, and its target protein ZEB2 was reduced, with significantly increased expression of its downstream proteins P-AKT (Ser473), PI3K p85α, Psmad3 (Ser425), and COL4 α1 in the glomeruli of DN mice (PI3K/AKT signaling pathway). This pathway is thought to play an important role in renal fibrosis. They found that metformin significantly decreased the expression of miR-192’s downstream proteins P-AKT (Ser473), PI3K p85, and Psmad3 (Ser425) in the glomeruli of DN mice. This suggests that the anti-renal-fibrosis activity of metformin may be related to decrease the expression of miR-192, increased expression of ZEB2 and inhibition of the PTEN/PI3K/AKT pathway.

In the current study, the Group I normoalbuminuric group had a significantly higher level of miRNA-192 (1.35 ± 0.5) than Group II (0.65 ± 0.2, *p*3-0.001) and Group III (normal healthy control group) (0.83 ± 0.3, *p*1-0.001).

In contrast to our study, Al-Kafaji et al. [23] reported that miR-192 expression was decreased 14-fold overall in T2DM patients with and without DN in comparison with the healthy control group (*p* < 0.005). The difference in the results may be attributed to the following differences: in our study, the age of the participating population was younger and matched with no statically significant difference (*p*-0.47) among the groups, while in Al-Kafaji et al. [23]’s study, the mean age was significantly higher in the diabetic patients compared with the healthy controls (*p* <0.05) and an older average age in their normoalbuminuric group of 60.3 ± 12.2 years compared with 51.8 ± 7.37 years in the normoalbuminuric group in our study. Additionally, the duration of DM of the normoalbuminuric group was 15.0 ± 4.4 years in Al-Kafaji et al. [23]’s study, compared with 5.23 ± 2.8 years in our study. Finally, Al-Kafaji et al. [23]’s study included a smaller sample size than our study.

On the other hand, Ma et al. [11] found that there was an insignificantly lower level of miR-192 expression in their normoalbuminuria group compared with their normal healthy control group (*p* > 0.05), which is different than our results.

In the current study, miR-192 plays a role in protection against nephropathy in diabetic patients without albuminuria and with eGFR > 60 mL/min/1.73 m^2^, showing a sensitivity of 56% and a specificity of 96% in comparison with the control subjects. It was also shown that miR-192 could tell the difference between diabetic patients with and without nephropathy, with a sensitivity of 90% and a specificity of 74%. Finally, miR-192 could distinguish the difference between diabetic patients with DN and the control group, with sensitivity of 82% and a specificity of 58%. This suggests that miR-192 may be a useful biomarker for finding people who have early-stage DN.

Wan et al. [8] conducted a literature review based on 28 studies; they concluded that miR-192 levels tend toward an increase in renal and urine tissues but decrease in serum in the case of diabetic nephropathy. The miR-192 serum level was downregulated in patients with DN in six out of eight trials (75%). On the other hand, most of the experimental studies conducted on cell or animal models of DN, 14 out of 18 (78%) studies in all, found that miR-192 was upregulated in the renal tissues. The authors explained this by the following hypothesis: the small molecules that present in the blood as miR-192 may deposit in renal tissue or encapsulate in renal fibrosis in the kidneys during their passage, leading to a high level of miR-192 in the renal tissues and subsequently, increased excretion in the urine. Ma et al. [11] suggested that the discrepancy in these studies may be due to differences in animal species, cell types, and experiment conditions.

Wan et al. [8] suggested that low-serum miR-192 might be helpful in the early diagnosis of DN, while a higher level of mi-192 in urine and renal tissues may suggest DN progression. Histopathological examination is the gold standard for identifying diseases. However, evidence shows the promising results of non-invasive methods (i.e., urinary and serum) in the early detection of DN. So, circulating miR-192 might represent a novel, noninvasive biomarker for DN; more studies should be designed to validate this finding.

Conflicting results might be attributed to different conditions, such as the miR-192 present in many variable tissues. There are many factors that affect miR-192 expression, including chronic medical conditions such as hypertension, medications like metformin, angiotensin-converting enzyme inhibitors (ACEIs), angiotensin receptor blockers (ARBs), and insulin [28] Additionally, physical activity may affect miR-192 expression [29] Most other studies overcame these factors that may affect miR-192 expression.

Oghbaei et al. [29] found that moderate activity for two months significantly increased the expression of miR-192 in in diabetic rats’ kidneys compared with the healthy rat group, while exercise significantly downregulated the miR-192 expression in the kidneys of healthy rat group compared with the control group.

Keyhanmanesh et al. [28] conducted an experimental study on a rat model of diabetes and found that the miR-192 expression significantly increased in the DN group. Insulin administration significantly decreased the expression levels of miR-192.

The limitations of this study are its short duration and the small number of patients, which could be affected by some medications such as insulin, metformin, which in turn could affect proteinuria such as angiotensin from converting enzyme inhibitors ACEIs and ARBs. The patients’ relative levels of physical activity could also affect the results. This study needs to be supported by a longer-duration study on the different stages of DN on a larger number of patients. We are working on another follow-up study regarding cases of diabetes without albuminuria and will discuss what happened to them and the percent of developed DN with an estimation of changes in miR-192 levels that can enforce their role in the early diagnosis and follow-up of DN.

## 5. Conclusions

This study highlights the potential role of serum miR-192 as a noninvasive biomarker for the early detection of DN in patients with type 2 DM. Our findings demonstrated that serum miR-192 levels were significantly reduced in patients with DN compared with diabetic patients without nephropathy and the healthy controls, suggesting a possible protective role of miR-192 in early disease stages. Despite the variability and controversy in the existing literature regarding miR-192 expression across different sample types and disease stages, our data align with several clinical and experimental studies supporting its diagnostic relevance. The significant correlation between the miR-192 levels, eGFR, and disease duration further reinforces its clinical utility. However, given the limited sample size and the short duration of the current study, larger longitudinal studies across different DN stages are essential to validate these findings and establish standardized protocols for the clinical application of miR-192 as a reliable biomarker in DN.

## Figures and Tables

**Table 1:** Demographic and biochemical findings in the studied groups.

	Group III (*n* = 50)	Group I (*n* = 50)	Group II (*n* = 50)	*p*
Sex				0.58
Male	34	29	32	
Female	16	21	18	
Age (years)	51.8 ± 7.37	52.28 ± 6.15	53.34 ± 5.47	0.47
BUN (mg/dL)	26.38 ± 2.77	27.68 ± 2.83	56.8 ± 4.73	0.001
Creatinine (mg/dL)	0.92 ± 0.2	1.02 ± 0.13	4.1 ± 0.6	0.001
Fasting blood glucose (mg/dL)	90.1 ± 11.4	150.4 ± 40.4	158.2 ± 60.4	0.001
HB1c (%)	4.4 ± 0.6	8.9 ± 2.01	8.5 ± 1.8	0.001
Duration (years)		5.23 ± 2.8	7.7 ± 3.3	0.001
Albumin (gm/dL)	4.04 ± 0.45	3.9 ± 0.2	3.7 ± 0.35	0.001
Albumin in urine (mg/g)	11.0 ± 4.9	17.01 ± 3.6	83.3 ± 16.6	0.001
Urinary creatinine (gm/24 h)	1. 71 ± 0.4	1.1 ± 0.3	0.7 ± 0.13	0.001
Albumin/creatinine ratio	12.5 ± 7.4	16.0 ± 1 5.0	117.6 ± 27.23	0.001
eGFR (mL/min/1.73 m^2^)	90.2 ± 19.2	69.6 ± 10.33	17.3 ± 4.02	0.001

BUN: blood urea nitrogen. eGFR: estimated glomerular filtration rate.

**Table 2:** Comparison of MicroRNA-192 among the studied groups.

MicroRNA-192	Group III (*n* = 50)	Group I (*n* = 50)	Group II (*n* = 50)	*p*
MicroRNA-192 (folds)	0.83 ± 0.3	1.35 ± 0.5	0.65 ± 0.2	*p*-0.001 *p*1-0.004 *p*2-0.001 *p*3-0.001

**Table 3:** Correlations among different clinical and biochemical parameters and MicroRNA-192.

	MicroRNA-192
Creatinine	
R	−0.651
P	0.001
eGFR	
R	0.744
P	0.001
Urinary creatinine	
R	0.488
P	0.001
Urinary albumin	
R	−0.616
P	0.001
Duration	
R	−0.302
P	0.001
Urinary albumin/creatinine ratio	
R	−0.624
P	0.001
eGFR	
R	0.354
P	0.000
BUN	
R	−0.419
P	0.000

eGFR: estimated glomerular filtration rate. BUN: blood urea nitrogen.

## Data Availability

The datasets generated and analyzed during the current study are available in the figshare repository at https://doi.org/10.6084/m9.figshare.25999315.v1 (accessed on 4 August 2024).

## References

[1] Shahin D.H.H., Sultana R., Farooq J., Taj T., Khaiser U.F., Alanazi N.S.A., Alshammari M.K., Alshammari M.N., Alsubaie F.H., Asdaq S.M.B. (2022). Insights into the uses of traditional plants for diabetes nephropathy: A review. Curr. Issues Mol. Biol..

[2] Shahbazian H., Rezaii I. (2013). Diabetic kidney disease; review of the current knowledge. J. Ren. Inj. Prev..

[3] Dronavalli S., Duka I., Bakris G.L. (2008). The pathogenesis of diabetic nephropathy. Nat. Clin. Pract. Endocrinol. Metab..

[4] Hu C., Sun L., Xiao L., Han Y., Fu X., Xiong X., Xu X., Liu Y., Yang S., Liu F. (2015). Insight into the mechanisms involved in the expression and regulation of extracellular matrix proteins in diabetic nephropathy. Curr. Med. Chem..

[5] Lutale J.J., Thordarson H., Abbas Z.G., Vetvik K. (2007). Microalbuminuria among type 1 and type 2 diabetic patients of African origin in Dar Es Salaam, Tanzania. BMC Nephrol..

[6] Gheith O., Othman N., Nampoory N., Halimb M., Al-Otaibi T. (2016). Diabetic kidney disease: Worldwide difference of prevalence and risk factors. J. Nephropharmacol..

[7] Tervaert T.W., Mooyaart A.L., Amann K., Cohen A.H., Cook H.T., Drachenberg C.B., Ferrario F., Fogo A.B., Haas M., de Heer E. (2010). Pathologic classification of diabetic nephropathy. J. Am. Soc. Nephrol..

[8] Wan X., Liao J., Lai H., Zhang S., Cui J., Chen C. (2023). Roles of microRNA-192 in diabetic nephropathy: The clinical applications and mechanisms of action. Front. Endocrinol..

[9] Arora M.K., Singh U.K. (2013). Molecular mechanisms in the pathogenesis of diabetic nephropathy: An update. Vasc. Pharmacol..

[10] Chang A.S., Hathaway C.K., Smithies O., Kakoki M. (2016). Transforming growth factor-β1 and diabetic nephropathy. Am. J. Physiol.-Ren. Physiol..

[11] Ma X., Lu C., Lv C., Wu C., Wang Q. (2016). The expression of miR-192 and its significance in diabetic nephropathy patients with different urine albumin creatinine ratio. J. Diabetes Res..

[12] Martínez-Hernández R., Marazuela M. (2023). MicroRNAs in autoimmune thyroid diseases and their role as biomarkers. Best Pr. Res. Clin. Endocrinol. Metab..

[13] Krattinger R., Boström A., Schiöth H.B., Thasler W.E., Mwinyi J., Kullak-Ublick G.A. (2016). 13microRNA-192 suppresses the expression of the farnesoid X receptor. Am. J. Physiol. Gastrointest. Liver Physiol..

[14] Mishan M.A., Tabari M.A.K., Parnian J., Fallahi J., Mahrooz A., Bagheri A. (2020). Functional mechanisms of miR-192 family in cancer. Genes Chromosomes Cancer.

[15] Ren F.J., Yao Y., Cai X.Y., Fang G.Y. (2021). Emerging role of MiR-192-5p in human diseases. Front. Pharmacol..

[16] Kidney Disease: Improving Global Outcomes (KDIGO) Diabetes Work Group (2020). KDIGO 2020 Clinical Practice Guideline for Diabetes Management in Chronic Kidney Disease. Kidney Int..

[17] Jiang F., Li C., Han J., Wang L. (2020). Diagnostic Value of Combination of MicroRNA-192 in Urinary Sediment and B-Ultrasound for Bladder Cancer. Technol. Cancer Res. Treat..

[18] Elsayed A.G.A., Badr D.F., El Kheir N.Y.A., Zaki M.E.S., Mossad A.E.M., Mahmoud E.M.F. (2024). Prevalence of extended-spectrum beta-lactamase and molecular detection of blaTEM, blaSHV, and blaCTX-M genotypes among gram-negative Bacilli isolates from hospital acquired infections in pediatrics, one institutional study. Ital. J. Pediatr..

[19] (2025). MedCalc Software Ltd., Ostend, Belgium. https://www.medcalc.org.

[20] DeLong E.R., DeLong D.M., Clarke-Pearson D.L. (1988). Comparing the areas under two or more correlated receiver operating characteristic curves: A nonparametric approach. Biometrics.

[21] Akpınar K., Aslan D., Fenkçi S.M., Caner V. (2022). miR-21-3p and miR-192-5p in patients with type 2 diabetic nephropathy. Diagnosis.

[22] Yang X., Liu S., Zhang R., Sun B., Zhou S., Chen R., Yu P. (2017). Microribonucleic acid-192 as a specific biomarker for the early diagnosis of diabetic kidney disease. J. Diabetes Investig..

[23] Al-Kafaji G., Al-Muhtaresh H.A. (2018). Expression of microRNA-377 and microRNA-192 and their potential as blood-based biomarkers for early detection of type 2 diabetic nephropathy. Mol. Med. Rep..

[24] Abou Eleila J.G., Abdel Wahab Mohamed A., Waked E.A., Kamel L.N., Amin H.S., Elhanafi H.M. (2024). Evaluation of serum MicroRNA 21, MicroRNA 192 and serum TGFβ1 in type 2 diabetes mellitus patients and their relation to diabetic nephropathy. Egypt. J. Med Hum. Genet..

[25] Chien H.Y., Chen C.Y., Chiu Y.H., Lin Y.C., Li W.C. (2016). Differential microRNA profiles predict diabetic nephropathy progression in Taiwan. Int. J. Med. Sci..

[26] Ebadi Z., Moradi N., Kazemi F.T., Balochnejadmojarrad T., Chamani E., Fadaei R., Fallah S. (2019). Captopril and spironolactone can attenuate diabetic nephropathy in wistar rats by targeting microRNA-192 and microRNA-29a/b/c. DNA Cell Biol..

[27] Yu S., Zhao H., Yang W., Amat R., Peng J., Li Y., Deng K., Mao X., Jiao Y. (2019). The alcohol extract of coreopsis tinctoria nutt ameliorates diabetes and diabetic nephropathy in db/db mice through miR-192/miR-200b and PTEN/AKT and ZEB2/ECM pathways. BioMed Res. Int..

[28] Keyhanmanesh R., Hamidian G., Lotfi H., Zavari Z., Seyfollahzadeh M., Ghadiri A., Ahmadi M., Bahari F., Bavil F.M. (2022). Troxerutin affects nephropathy signaling events in the kidney of type-1 diabetic male rats. Avicenna J. Phytomed..

[29] Oghbaei H., Asl N.A., Sheikhzadeh F., Alipour M.R., Khamaneh A.M. (2015). The effect of regular moderate exercise on miRNA-192 expression changes in kidney of streptozotocin-induced diabetic male rats. Adv. Pharm. Bull..

